# Updates on Triple-Negative Breast Cancer in Type 2 Diabetes Mellitus Patients: From Risk Factors to Diagnosis, Biomarkers and Therapy

**DOI:** 10.3390/diagnostics13142390

**Published:** 2023-07-17

**Authors:** Sabine Matou-Nasri, Maram Aldawood, Fatimah Alanazi, Abdul Latif Khan

**Affiliations:** 1 Blood and Cancer Research Department, King Abdullah International Medical Research Center (KAIMRC), King Saud bin Abdulaziz University for Health Sciences (KSAU-HS), Ministry of National Guard Health Affairs (MNG-HA), Riyadh 11481, Saudi Arabia; 442204103@student.ksu.edu.sa (M.A.); falanaz4@gmu.edu (F.A.); 2 Biosciences Department, Faculty of the School for Systems Biology, George Mason University, Manassas, VA 22030, USA; 3 Post Graduate and Zoology Department, King Saud University, Riyadh 12372, Saudi Arabia; 4 Tissue Biobank, KAIMRC, MNG-HA, Riyadh 11481, Saudi Arabia; khanab4@mngha.med.sa; 5 Pathology and Clinical Laboratory Medicine, King Abdulaziz Medical City (KAMC), Riyadh 11564, Saudi Arabia

**Keywords:** triple-negative breast cancer, type 2 diabetes mellitus, risk factors, biomarkers, diagnosis, prognosis, therapy

## Abstract

Triple-negative breast cancer (TNBC) is usually the most malignant and aggressive mammary epithelial tumor characterized by the lack of expression for estrogen receptors and progesterone receptors, and the absence of epidermal growth factor receptor (HER)2 amplification. Corresponding to 15–20% of all breast cancers and well-known by its poor clinical outcome, this negative receptor expression deprives TNBC from targeted therapy and makes its management therapeutically challenging. Type 2 diabetes mellitus (T2DM) is the most common ageing metabolic disorder due to insulin deficiency or resistance resulting in hyperglycemia, hyperinsulinemia, and hyperlipidemia. Due to metabolic and hormonal imbalances, there are many interplays between both chronic disorders leading to increased risk of breast cancer, especially TNBC, diagnosed in T2DM patients. The purpose of this review is to provide up-to-date information related to epidemiology and clinicopathological features, risk factors, diagnosis, biomarkers, and current therapy/clinical trials for TNBC patients with T2DM compared to non-diabetic counterparts. Thus, in-depth investigation of the diabetic complications on TNBC onset, development, and progression and the discovery of biomarkers would improve TNBC management through early diagnosis, tailoring therapy for a better outcome of T2DM patients diagnosed with TNBC.

## 1. Introduction

Diabetes is a serious metabolic disorder characterized by sustained hyperglycemia, hyperinsulinemia, and hyperlipidemia due to insulin resistance, which potentially enhances the risk of cancer, and especially triple-negative breast cancer (TNBC) in women [1]. Diabetes and cancer are two major public health problems occurring mainly in the U.S, European nations, and emerging developed countries [2]. As per the Centers for Disease Control and Prevention, in these modern countries with upper-middle incomes cancer is the second and diabetes is the seventh leading cause of death due to life-threatening complications, including vascular complications, heart disease, and cancer, recently predicted to become the leading cause in diabetes [3,4]. In 2021, over 537 million people (aged 20–79 years) were reported to have diabetes mellitus worldwide with a predicted rise to 643 million and 783 million by 2030 and 2045, respectively [5]. Because of a rich life, deficient exercise, and undesirable dietary propensities, around 90% of these individuals have type 2 diabetes mellitus (T2DM, the most common form of diabetes) leading to an intensified health economic burden predicted to rise from USD 1.3 trillion in 2015 to USD 2.1 trillion by 2030 [6,7,8]. Of note, the cost of treatment considerably increases threefold to ninefold when the patient presents diabetes-related complications such as retinopathy, cardiovascular diseases, and end-stage renal disease versus those without [9,10]. Numerous epidemiological studies have reported that T2DM patients have 20–30% higher risk of developing TNBC, the advanced stage responsible for 50% of breast cancer mortality [1,11,12]. At the economic level, TNBC is associated with a substantial economic burden on the healthcare system [13]. In addition, patients who have both diabetes and TNBC result in worse patient-reported outcomes due to the high risk of distant relapse [1,14]. The number of the mortality rate due to TNBC has been projected to increase in East and South Asian countries in the next ten years [15]. Previous meta-analysis studies have reported that having diabetes is associated with a 20–30% increased mortality risk among cancer patients under insulin therapy with long diabetes duration, which was more apparent among liver, breast, endometrial, and colorectal cancer patients [16]. Through a cross-sectional study coupled with tumor tissue micro-array analysis, T2DM patients with breast cancer have been reported to develop more hormone receptor-negative breast tumors such as TNBC than other breast cancer subtypes [17,18,19]. In addition, breast cancer in postmenopausal women with T2DM is often diagnosed at an advanced stage corresponding mainly to TNBC, and they are at higher risk of invasive cancer and cancer-related death than non-diabetic counterparts [20]. The anti-diabetic treatment of cancerous patients with metformin, the first line anti-hyperglycemic drug with anti-cancer effects, can only prevent and reduce the cancer but presents limitations in the eradication of highly invasive cancer tissues even when combined with neo-adjuvant chemotherapy [20,21]. Thus, there is an urgent need to establish preventive medicine, precise cancer diagnosis, and a novel targeted therapeutic approach in order to improve diabetes management and to enhance cancer cell sensitivity to chemotherapy and radiotherapy to treat TNBC, which will also reduce the healthcare cost of the diabetic patients diagnosed with cancer. This review emphasizes on the risk factors for TNBC development and progression on TNBC subtypes at the molecular and transcriptional levels, on current and potential biomarkers, therapy, and ongoing clinical trials for T2DM patients versus their non-diabetic counterparts.

## 2. Epidemiology—TNBC Risk in T2DM Patients

### 2.1. TNBC, the Major Breast Cancer Subtype in T2DM Patients

Patients with T2DM have an increased risk of developing TNBC, a heterogeneous group of breast carcinomas with most of them characterized by a fast-growing and aggressive subtype with different genetic profiles. Some rare TNBC cases are low-grade and poorly invasive with an indolent clinical outcome; however, TNBC is frequently diagnosed at a later stage, associated with a larger tumor size, a higher tumor grade, poorly differentiated invasive ductal carcinoma histological subtype, increased rate of lymph metastasis, and associated with a poor 5-year survival [19,22]. TNBC is often characterized by early systemic relapse. High proliferative breast cancers such as TNBC are more likely to be missed in regular screening [1]. Early detection of TNBC is crucial as TNBC rapidly grows and spreads, although responding well to neoadjuvant chemotherapy, few TNBCs that respond less reoccur sooner. In addition, invasive TNBCs present high potential for metastases, particularly in the lungs and the brain. TNBC is also a rapidly evolving disease, associated with young age, and shows benign features, including an oval or round shape, a smooth or circumscribed margin, and is less likely to have an echogenic halo as a sign of malignancy [23].

### 2.2. TNBC Subtypes

TNBC, a highly invasive heterogeneous subtype with the poorest outcome, is clinically and molecularly defined by the lack of estrogen receptor (ER-negative or ER^−^) and progesterone receptor (PR-negative or PR^−^) expression and the absence of human epidermal growth factor receptor 2 (HER2-negative, HER2^−^) overexpression detected by immunohistochemistry (IHC) staining. However, recently, a negligible percentage corresponding to less than 1% of ER and PR expression has been detected [24]. Among all the breast cancer cases, one woman out five or even six develops TNBC [25]. Using magnetic resonance imaging in 1090 women (mean of 52.1 years) of whom 256 were diagnosed with TNBC and 846 were diagnosed with ER-positive, the TNBC was revealed to be closer to the chest with a tendency to develop towards a posterior or prepectoral location compared with the ER-positive breast cancers [26]. An in-depth knowledge of the TNBC at the molecular level enables the establishment of rational stratification reflecting intrinsic and clinical differences between subtypes in response to various therapies to sharpen the treatment approaches for better clinical outcomes [25,27,28,29]. Thus, the clinical relevance of the TNBC subtype classification has been correlated with chemotherapeutic response and eventually to the prediction of the tumor pathologic complete response (pCR) [30,31]. Based on transcriptome expression profile analysis of invasive carcinomas and their drug sensitivity, Lehmann and colleagues established the classification of TNBC into seven distinct clusters also named as subtypes, including one uncharacterizable unstable (UNS) subtype and six well-characterized stable subtypes [32]. The transcriptional analysis of the stable subtypes revealed two basal-like (BL1 and BL2) subtypes harboring proliferation genes, an immune-modulatory (IM) subtype enriched with immune-associated genes, a mesenchymal (M) subtype characterized by cell motility, a mesenchymal stem-like (MSL) subtype highly expressing angiogenesis- and stemness-related genes, and a luminal-androgen receptor (LAR) subtype sensitive to androgen activity and enriched in HER2 expression (Table 1). Considering extrinsic signals emitted from immune and stromal cells, Brown’s group (2015) classified 198 breast tumors into only four TNBC subtypes, which are BL-immunosuppressed, BL-immunoactivated, MSL, and LAR based on genomic analysis [33]. Lehmann and colleagues also refined the TNBC classification to TNBCtype-4 (BL1, BL2, M, and LAR) based on lymphocyte infiltration, stromal mesenchymal cell gene expression, and the response to neoadjuvant chemotherapy [34]. At the diagnosis, the BL (expressing marker of the myoepithelium of the normal gland) is revealed as the major TNBC subtype, representing 80% of all subtypes while 15% of TNBCs are the LAR subtype [35].

### 2.3. Genetic Mutations in TNBC Subtypes

The main genetic mutations, reported to be strongly associated with TNBC development, are breast cancer genes. Both *BRCA1/2* mutations are majorly detected in BL1 while the *BRCA1* mutation is also detected in the BL1 and MSL TNBC subtypes [36]. The tumor suppressor *TP53*, phosphatase and tensin homolog (*PTEN*), and cyclin-dependent kinase inhibitor 2A (*CDKN2A*) gene mutations are often found in BL1, BL2, and ML subtypes while the LAR TNBC subtype harbors *TP53*, *PTEN*, and phosphatidylinositol-4,5-bisphosphate 3-kinase catalytic subunit alpha (*PIK3CA*) gene mutations. Considering TNBC clinical behavior, prognosis, and multidrug resistance, a PTEN-reduced/PI3K-high/mammalian target of rapamycin (mTOR)-high expression is suggested to constitute a high-risk profile of TNBC progression [37]. The metastatic MSL TNBC subtype is mainly characterized by mutations in angiogenesis-related genes such as *HRAS*, *KRAS*, and platelet-derived growth factor receptor-alpha (*PDGFRA*). The two other TNBC subtypes contain genomic alterations resulting in mutations in immune-related genes such as nuclear factor kappa-B inhibitor alpha (*NFKBIA*) and tumor suppressor *APC* [38]. Added to the characterization of the TNBC subtypes, the related signaling pathways, genetic markers, and potential therapy are summarized in the Table 1.

### 2.4. Risk Factors

Although the specific etiology of TNBC has not been described, many predisposing risk factors such as gender; age; genetic mutations and family history; reproductive history; ethnicity; and precipitating risk factors, including body mass index (BMI), physical inactivity, sex hormonal imbalances, vitamin supplements, alcohol intake and smoking, and metabolic syndrome, have led to TNBC occurrence [39]. While T2DM is an independent risk factor of TNBC, the main T2DM-related risk factors associated with TNBC onset and development are age, high BMI (i.e., overweight and obesity), and physical inactivity.

**Table 1:** Characterization of triple-negative breast cancer subtypes according to Lehmann classification.

TNBC Subtype	Signaling PATHWAYS	Genetic Markers	Potential Therapy
**Basal-like 1 (BL1)**	Cell cycle, DNA damage response [32]	BRCA1/2; TP53; PTEN; CDKN2A [37]	PARP inhibitors, Mitosis inhibitors, Cytostatics, DNA Synthetic inhibitors [25,28,29]
**Basal-like 2 (BL2)**	Growth factor-signaling pathways (NGF, EGFR, MET, IGF-1R, Wnt/β-catenin), gluconeogenesis and glycolysis pathways [32]	BRCA1; TP53; CDKN2A; PTEN [37]	PARP inhibitors, Cytostatics, Growth Factor inhibitors, mTOR inhibitors [25]
**Immune-modulatory (IM)**	Cytokine signaling, β-catenin signaling, immune cell process pathway, antigen processing and presentation, and signaling pathways [32,38]	NFKBIA; APC; JAK1/2, STAT1/4, IRF1/7/8, TNF [36,38]	Immune checkpoint inhibitors, PARP inhibitors, Cytostatics [25]
**Mesenchymal-like (ML)**	Epithelial-mesenchymal transition (EMT), cell proliferation, cell motility, and growth factor signaling pathways [27]	TP53; CDKN2A; PTEN; PIK3CA [37]	Src inhibitors, mTOR inhibitors, PI3K inhibitors, Growth Factor inhibitors [25,29]
**Mesenchymal stem-like (MSL)**	Epithelial-mesenchymal transition (EMT), transforming growth factor (TGF)-beta, extracellular matrix (ECM)-receptor interaction and focal adhesion signaling pathway, Angiogenesis genes, low proliferation [27,32]	BRCA1; HRAS, KRAS; PDGFRA [37]	Src inhibitors, mTOR inhibitors, PI3K inhibitors, Growth Factor inhibitors, MAPK inhibitors [25,29]
**Luminal androgen receptor (LAR)**	Steroid hormone biosynthesis, porphyrin and chlorophyll metabolism, androgen and estrogen metabolism, peroxisome proliferator-activated receptor (PPAR) signaling pathway [27,32]	TP53; PTEN; PIK3CA; HER2 [37]	PI3K inhibitors, mTOR inhibitors, Nonsteroidal antiandrogens [25,29]

## 3. Predisposing Risk Factors

### 3.1. Gender

The main predisposing risk factor for all types of breast cancer including TNBC is the gender as women are more predisposed to develop breast cancer than men, who represent only 0.02 percent of all breast cancer cases [40]. BRCA1/2 mutation carriers including BRCA2 mutation are found in 14–40% of all male TNBC cases [41]. The TNBC risk also increases in men due to family history, infertility, Klinefelter’s syndrome, smoking, altered estrogen-testosterone ration, high androgen and estrogen levels, and previous thoracic radiotherapy [41]. The male breast cancer is usually detected as a hard lump underneath the nipple and areola [42]. A US population-based concluded that male TNBC management has been challenging as male TNBC likely to present more advanced stage compared to their female counterparts and request further pre-clinical research despite the small number of cases in order to improve the overall survival in men with TNBC [43].

### 3.2. Age

The age factor is a crucial parameter in TNBC onset and development as TNBC risk increases with the age. The median age of women presenting TNBC is around 60, much younger than those diagnosed with other breast cancer subtypes [44,45]. The median age of men diagnosed with TNBC is around 70–75 [41]. A recent clinical observation concluded that the young age should be considered as an additional adverse prognostic feature in therapeutic management of TNBC [44]. A comparative clinical study performed in various age groups of Swedish patients with TNBC reported that primary TNBC in younger patients is often of a poor differentiation grade and more highly proliferative than older counterparts, suggesting a tailored treatment [46]. T2DM and TNBC share risk factors such as older age. In an observational epidemiological study dealing with Chinese T2DM patients diagnosed with TNBC, the mean of age was above 50, including 56.9 ± 11.2 years for (104) T2DM patients and 50.48 ± 11.0 years for (761) non-T2DM patients [47]. A retrospective case–control study conducted from the Louisiana Tumor Registry records of primary invasive TNBC diagnosed in 2010–2015 revealed a significant association between T2DM and breast cancer for Luminal A and TNBC subtypes diagnosed in both African American and non-Hispanic White women aged above 50 years [18]. However, for most of the epidemiological studies, comparing breast cancer women diagnosed with and without T2DM, the age of the participants was quite similar in both groups [1,17].

### 3.3. TNBC and T2DM-Shared Susceptibility Genes

Several genetic-based studies, especially large-scale, genome-wide association studies (GWAS), have been conducted to understand the etiology of complex diseases such as breast cancer and T2DM. Large-scale GWAS of T2DM and breast cancer have provided evidence for shared susceptibility genes, including a fat mass and obesity-associated gene (*FTO*), the interleukin-6 (*IL-6*) gene, and heat-shock protein 60 encoded by *HSPD1* gene [48]. Genomic DNA samples extracted from blood samples of 1168 breast cancer cases of T2DM Chinese females were genotyped for three diabetes-related single nucleotide polymorphisms (SNPs) genetic factors *FTO* rs3751812, *IL-6* rs1800796, and *HSPD1* rs2605039. It was revealed that T2DM individuals with CC genotype of *IL-6* rs1800796 or GG genotype of *HSPD1* rs2605039 were strongly associated with increased risk of breast cancer (OR (95%CI): 2.53 (1.45, 4.41) and OR (95%CI): 6.40 (2.29, 17.87), respectively) compared to non-T2DM individuals [48]. A better progression free survival for breast cancer patients was shown to be associated with GT/TT genotypes of *HSPD1* rs2605039, suggesting *HSPD1* influence on breast cancer survival [48]. The upregulation and key role of FTO in TNBC survival and tumorigenic potentials was revealed by facing triple-negative inflammatory BC cell line SUM149 to severe metabolic challenges after glutamine deprivation [49]. As both T2DM and TNBC are inflammatory diseases, an in vitro study reported the important role of the pro-inflammatory IL-6 signaling in TNBC survival and aggressiveness, suggesting IL-6 inhibition as a potential therapeutic intervention and/or new adjuvant therapy to improve the clinical outcome of TNBC patients [50]. High levels of Hsp60 in the plasma and saliva of T2DM patients were detected compared to non-diabetic controls and immunohistochemical analysis in 66 TNBC tissues of Jordanian women that revealed that Hsp60, considered as an inflammatory marker of T2DM, was highly expressed in advanced stage of the tumor, considering Hsp60 as a poor prognosis marker in TNBC patients [51,52,53].

Additional genetic polymorphisms-related studies reported more diabetes-related risk SNPs (i.e., solute carrier family 30 member 8 *SLC30A8*-rs4876369 and insulin receptor substrate 2 *IRS2*-rs2241745) associated with first primary invasive breast cancer risk in 817 Caucasian women while other SNPs (i.e., cyclin-dependent kinase inhibitor *CDKN2A/CDKN2B*-rs3218020, Cdk5 regulatory subunit-associated protein 1-like 1 *CDKAL1*-rs981042, transcription factor 2/Hepatocyte nuclear factor-1-beta *TCF2/HNF1B*-rs3094508) were strongly associated with breast cancer-specific mortality [54]. Predominantly expressed in pancreatic islets of β-cells, the *SLC30A8* gene encodes zinc transporter ZnT-8 and its variants were correlated with improved glucose tolerance, protection from T2DM development, and reduction of T2DM risk [55]. Using luminal-like and basal-like TNBC, gene expression profiling of Zn transporters, including ZnT-8, differed according to the breast cancer subtype [56,57]. Luminal low-invasive T47D accumulated more Zn transporters than basal-like highly invasive MDA-MB-231 cells, suggesting an inverse correlation of Zn transporter expression with the malignant phenotype in breast cancer [57]. Playing a key role in the control of glucose homeostasis, a downregulation of insulin signaling adaptor protein IRS2 was monitored in the liver of obese individuals with T2DM compared to non-diabetic counterparts [58]. A genomic and transcriptomic-based study reported that the use of whole exome sequencing on primary TNBC and paired blood samples from 465 female Chinese patients revealed somatic copy number alterations (CNAs) resulting in frequent gains in *IRS2* gene, observed in 49% of all TNBC patients [59]. At the tissue level, a higher expression level of IGF1R but no change in IRS2 expression level was found in the negative HER2-breast cancer of patients with T2DM compared to non-diabetic counterparts [60]. Examining the frequency of CNAs in 32 known breast cancer copy number driver genes in the whole TNBC cohort, the BL1 subtype presents the highest number of CNAs with high gain/amplification levels involving *CDKN2A/B* (FDR = 5.6 × 10^−5^ and 7.5 × 10^−5^) genes than other subtypes, providing the basis for future genomic-driven targeted therapies [61]. *CDKAL1*, mainly reported as a susceptibility gene for T2DM through sustained systemic glucose homeostasis and adipose mitochondrial function regulation, has been linked to luminal-AR TNBC subtype [33,62]. The other susceptible gene causing maturity-onset diabetes of the young (MODY) is the transcription factor *TCF2/HNF1B* involved in β-cell development and function, and is reported to be negatively associated with overall survival in breast cancer (HR = 1.69, *p* = 0.0044) [63,64]. The identification of polygenic risk factor or genetic polymorphisms that increase the risk of developing T2DM and the risk of TNBC would contribute to preventive medicine and enhance our understanding of how T2DM affects TNBC risk, which may be important for reducing the high burden of TNBC in T2DM patients.

### 3.4. Family History

Hereditary breast cancer, particularly the susceptible germline mutation *BRCA1/2*, is the major risk factor associated with breast cancer and corresponds to 5–10% of all breast cancers [65,66]. Several genetic syndromes such as hereditary breast and ovarian cancer (HBOC) syndrome have also been associated with familial breast cancer. Approximately 15–20% of all breast cancer cases are due to family history, indicating the involvement of inherited components in the development of some breast cancers. Having a first-degree relative diagnosed with breast cancer doubles the breast cancer risk. Among women with at least two affected first-degree relatives, risk of all the subtypes including TNBC and the predominant ER^+^ subtype, and ER^−^/PR^−^/HER2^+^ breast cancer was the most pronounced compared to women with no affected degree relatives [67]. Analyzing data from (10,549) patients with breast cancer, it was observed that patients with first-degree relatives diagnosed with breast cancer were older at diagnosis and presented breast cancer at a later stage than those with second/third-degree relatives [68]. There is an importance of preventive medicine through implementation of genetic screening high-risk groups [65]. After reviewing the medical histories of (6052) women with *BRCA1* or *BRCA2* mutation, half of them developed breast cancer (especially TNBC) and faced a twofold increase risk of diabetes in the 15 years after breast cancer diagnosis [69]. T2DM development was seen in BRCA1/2 carriers even in a mean of 8.6 years after breast cancer diagnosis due to the chemotherapy and high BMI value [69]. Primarily found within the nucleus, the main biological function of the tumor suppressor BRCA is the maintenance of genome stability through its functional interactions with DNA repair enzymes and demonstrated as a component of the RNA polymerase II holoenzyme, playing a role as transcriptional activator [70]. In addition, BRCA has also been described as a metabolic regulator through its complex interactions with insulin/IGF-1 signaling axis and as transcriptional repressor of the gene *IGF1R*, main key players in the pathogenesis of T2DM-related complications and in breast cancer onset and progression [68,70].

### 3.5. Reproductive History

TNBC etiology is also due to reproductive lifestyle such as early age at menarche, late menopause and remaining nulliparous (i.e., absence of pregnancy beyond 20 weeks), late age at first completed pregnancy, and lack of breastfeeding, increasing by twofold the risk of TNBC [71,72]. A recent Japanese clinical investigation considering clinicopathological characteristics and prognosis on primary (5153) invasive breast cancer cases with stage I-III reported that postmenopausal TNBC patients tend to have a better disease-free survival than premenopausal counterparts do [73]. Women who experience late menopause (aged after 55) have an increased risk of developing T2DM [74]. Although T2DM is the most common chronic disease in postmenopausal women (aged after 55), large Latin American, Korean, Indian, and American studies found that women aged under 45 and diagnosed with T2DM have higher risk of early menopause [75,76,77,78]. This early menopause onset has been suggested to be due to premature vascular aging caused by T2DM and subsequently accelerating ovarian aging [79]. However, other studies performed in the US based on the National Health and Nutrition Examination Survey reported that longer duration of T2DM delays the onset of menopause in women diagnosed aged 50 and above [80].

### 3.6. Ethnicity

Population-based analyses estimated that the annual average of TNBC incidence corresponds to 15 in 100,000 women, especially non-white premenopausal (less than 40 years) individuals developing TNBC [81]. Recent US and European epidemiological studies have reported that the highest TNBC-related mortality rate (i.e., 28%) affects mainly African American women and non-Hispanic white women diagnosed more than their White counterparts because of their income and lifestyle (i.e., unhealthy alcohol use and food intake) and to their disparities in receipt of surgery and chemotherapy [82,83,84]. Global epidemiological studies reported that TNBC prevalence is 12% in US patients while it is 2.7 times higher in African American patients [85], 12–25% in India [86], 27% in Africa (45.7% in West Africa and 14.9% in Central Africa) [87], 29.5% in the Chinese Southern Shaanxi Province [88], and an incidence of 30.4% in Saudi Arabia [89]. Based on prospective large US cohort of T2DM women, a racial disparity in the incidence of ER^−^ and TNBC was reported between European-American or non-Hispanic white women and African American women, indicating a 20% increased risk of developing breast cancer in T2DM White women while increased risks of 43% and 92% of ER^−^ development in T2DM African American women and their non-obese counterparts, respectively [62].

### 3.7. Breast Density

Breast density is often a hereditary trait, which is measured by mammography representing the volume of fibroglandular (dense) tissue relative to fatty (non-dense) tissue. Breast density can also increase by using menopausal hormonal therapy and having a low body mass index [90,91,92]. TNBC can present with benign imaging features on mammography, ultrasound, or magnetic resonance imaging such as benign fibroadenomas, making imaging features of TNBC crucial [45]. Dense breasts are more associated with TNBC risk in premenopausal women than in postmenopausal women. In addition, premenopausal women presenting with dense breasts have 2.8 times more risk of TNBC than their non-dense breasts counterparts do [93].

Several observational epidemiological studies based on measurements of breast density provided evidence that the association between T2DM and mammography density is differential depending on the type of diabetes treatment [94]. Another epidemiological study including the participation of Danish breast cancer postmenopausal women with 2.4% of these patients being diabetic, including T2DM, concluded that the control of diabetes by diet or oral antidiabetic agents decreases breast density while taking insulin increased breast density due to the mitogenic effect of insulin on mammary epithelial cells [94]. An increase in breast density was reported after a mammography screening-based study design performed on age-matched T2DM breast cancer patients after long-term insulin exposure [95]. Overall, T2DM is associated with breast density reduction, but an increase in breast cancer risk in postmenopausal women. Under insulin treatment, increased breast density in T2DM patients diagnosed with breast cancer (TNBC) can also complicate cancer screening and diagnosis [96].

## 4. Precipitating Risk Factors

### 4.1. Body Mass Index (BMI)

BMI is a common index derived from the weight and height of the individual, estimating the amount of body fat and indicating the healthy weight status of the person. Three categories have been classified based on BMI value such as healthy weight corresponding to an average of 23 kg/m^2^ while underweight, overweight, and obese subjects present BMI value lower than 18.5 kg/m^2^, in the range of 25–29.9 kg/m^2^, and greater than 30 kg/m^2^, respectively. However, it has been well documented that obesity is associated with decreased risk of premenopausal breast cancer and increased risk of postmenopausal breast cancer [97,98]. Due to the amount of fat tissue known to present a source for peripheral estrogen estrone (E1) production after menopause, BMI is positively associated with tissue levels of circulating estrone (E1) in postmenopausal women with increased risk of ER^+^ breast cancer and a reduced risk of TNBC [99,100,101,102]. Recent epidemiological studies reported that obesity and menopausal status are strongly associated with TNBC risk as premenopausal obese women have higher risk (42%) of developing TNBC than their non-obese counterparts [98]. Thus, in clinical practice, BMI is an important parameter measured at the time of diagnosis of TNBC.

### 4.2. Physical Inactivity

Based on epidemiological and observational studies conducted in large cohorts, recreational physical activities are one of the most modifiable factors to be encouraged to perform for reduction or even prevention of the risk of breast cancer, especially TNBC [103]. Previous studies reported the decreased risk of TNBC in postmenopausal women compared with counterparts who performed no physical activity [103,104]. This association between leisure-time physical activity and TNBC risk was recently observed in obese versus non-obese after age adjustment in premenopausal Nigerian women [104]. Physical activity and a healthy diet lower body fat, sex hormonal level, and adipose tissue cytokine production, and decrease inflammation, which improves quality of life in survivors of TNBC [105]. Promoting physically active lifestyles to reduce TNBC risk is of public health importance.

### 4.3. Sex Hormonal Imbalances

TNBC etiology may be also due to hormonal imbalances caused by oral contraceptives or menopausal replacement therapy. The existence of a strong link between hormonal imbalances and the risk of breast cancer development in women has been well defined unlike in men, as breast cancer like ovarian cancer is known as female hormone-responsive cancer. The long exposure of ovarian hormones, endogenous estrogen, and progesterone may cause breast cancer onset. Pre-clinical studies and observations suggested that estrogen might promote the development of ERα-negative breast cancer subtypes, including TNBC [12]. Although TNBC does not express ER and PR, hormone replacement therapy containing mainly estrogens and progestogens, and especially on the LAR subtype through their influence on androgens and interactions with ERβ receptors through AR:ERβ heterodimers, which may modulate the growth and invasiveness of TNBC [106,107]. Recently found to be expressed in approximately 18% of TNBC tissues, ERβ1 has been revealed to exhibit oncosuppressive activities and associated with favorable clinicopathological features but depends on menopausal status while its isoform variants ERβ2 and ERβ5 present pro-oncogenic properties [108,109]. Therefore, women diagnosed with TNBC who have more ERβ expressed tend to have higher overall survival than those with lower ERβ levels [110]. Promising research studies using a xenograft preclinical mouse model with human TNBC containing higher levels of ERβ reported greater reduction in TNBC growth and metastasis [111]. In addition, the selective treatment of TNBC cells with the chloroindazole compound mimicking the activating effects of the estradiol on ERβ and not on ERα confirmed the oncosuppressor role of ERβ activation [111]. Thus, the strict control and maintenance of hormonal equilibrium during the different life periods from the menarche through the menopause are important preventive strategies against breast cancer, including TNBC, in women. Sex hormonal imbalance characterized by increased levels of endogenous sex hormones correlates with increased BMI. High levels of testosterone are associated with increased risk of T2DM among women [112]. Recently, ERβ activation has been reported to be involved in insulin secretion, resistance and in glucose uptake, defining ER involvement in T2DM etiology and suggesting ERβ as a therapeutic target for T2DM management [113]. In addition, insulin-like growth factor (IGF), particularly IGF2 highly expressed in TNBC tissues, upregulates ERβ expression in TNBC [114,115]. Altogether, ERβ may be an interesting potential target for T2DM patients diagnosed with TNBC.

### 4.4. Vitamin Supplements

Most of the vitamins are well known for their anticancer properties that can impede TNBC development and progression. In vitro studies have reported the anticancer effects of vitamin C and vitamin B3-derived nicotinamide on TNBC development and progression by decreasing pro-angiogenic hypoxia-inducible factor HIF-1α and increasing pro-apoptotic reactive oxygen species levels, respectively [116,117]. The active metabolite of vitamin D, 1α,25-dihydroxyvitamin D_3_—also known as cholecalciferol, presents protective effects through its interactions with several proteins involved in cell growth arrest, DNA damage, and in the inactivation of cathepsin L-mediated degradation of p53 binding protein 1 (TP53BP1) [118]. Vitamin D deficiency is associated with insulin resistance leading to T2DM and may promote breast cancer risk in patients with T2DM [119,120,121]. Vitamin D can be found sequestered in adipose tissue, so BMI may affect vitamin D on TNBC risk. Epidemiological data indicated that vitamin D supplementation (2000 IU/d) is associated with lower TNBC incidence and higher survival rates of TNBC patients, especially among patients with dark skin [118]. Increased sun exposure and consequently enhanced vitamin D synthesis was associated with a reduced risk of TNBC in African American/women with dark skin [122]. Based on clinical intervention data, there are strong associations between the levels of the circulating form of vitamin D (hydroxyvitamin D, 25-OHD) and its nuclear receptor VDRnuc expression level and TNBC early stage, suggesting vitamin D as a prognostic biomarker in early TNBC [123,124]. In addition, 25-HOD insufficiency is prevalent in younger and obese TNBC patients [124].

### 4.5. Alcohol Intake and Smoking

Lifestyle, including alcohol intake and smoking, has a major impact on promoting breast cancer malignancy through the overproduction of estrogens, alterations in estrogen receptors, oxidative stress, DNA damage, genotoxicity, epithelial–mesenchymal transition promotion, and over-activation of epidermal growth factor receptor (EGFR)/ErbB2 signaling [125,126]. Although both unhealthy factors such as alcohol intake and smoking are strongly associated with breast cancer-related mortality rate and the risk of ER^+^ breast cancer, their association with TNBC risk is more controverted [127,128,129]. Epidemiological data of a large cohort of 146,985 postmenopausal women showed that drinkers have a reduced risk of developing TNBC compared to non-drinkers while TNBC incidence was not associated with cigarette smoking [130]. In contrast, a recent population health assessment conducted on 3449 TNBC patients from New Castle County (DE, USA) has reported that unhealthy alcohol use was strongly associated with high TNBC incidence [131]. In addition, another epidemiological study with a smaller cohort found a significant association of current smoking with the risk of TNBC while no association was obtained between former smoking and TNBC risk, suggesting that quitting smoking may reduce TNBC risk [132].

### 4.6. Metabolic Syndrome

Metabolic syndrome, a cluster of biological irregularities resulting in hypertension, dyslipidemia, obesity, insulin resistance, and subsequently diabetes, was reported to be a robust risk factor for TNBC in a case–control study of 555 West African women [133]. A retrospective study evaluating 1416 patients with T2DM diagnosed with breast cancer between 2015 and 2020 showed that poor glycemic control was strongly associated with the risk of TNBC development [14]. An Indian clinical study reported that TNBC patients who underwent seven-cycle neoadjuvant chemotherapy had presented more biological markers characteristic of metabolic syndrome including T2DM than non-treated TNBC patients [134]. In addition, TNBC patients were more subject to developing metabolic syndrome than triple-positive breast cancer patients, which reinforces the intricate relationship between T2DM and cancer, two complex chronic metabolic morbidities [134].

## 5. Diagnosis of TNBC in T2DM Patients

T2DM, essentially hyperglycemia and known as a common comorbidity of obesity, is considered an independent prognostic risk factor leading to an increased risk for TNBC onset, development, recurrence, and metastasis [19]. Many epidemiological studies reported that T2DM patients have an increased cause-specific mortality rates and decreased disease-free survival (DFS) rates compared with their non-diabetic counterparts, even after adjustment of other comorbidities and confounders, including age (>50 or ≤50 years), menopausal status, tumor size (>5 or ≤5 cm), lymph node involvement (positive or negative), and adjuvant chemotherapy status (chemotherapy or no chemotherapy). A small cohort of 865 Chinese patients diagnosed with primary invasive early stage of TNBC, including 104 postmenopausal T2DM patients, indicated that the DFS rates at 2 years were 78% for T2DM group with positive lymph nodes and 97% for non-T2DM group with no detection of lymph nodes [47]. However, one epidemiological study conducted in an Egyptian University dealing with clinicopathological data of 177 T2DM BC patients and 199 non-diabetic breast cancer patients did not find any differences regarding breast cancer subtypes [135]. The mean age of T2DM patients with breast cancer was 59 while the mean age of non-diabetic breast cancer patients was 48, and the only significant difference between both groups was the detection of more tumors in T2DM patients than in their non-diabetic counterparts [135]. A meta-analysis study reported that most of the epidemiological studies provided evidence that women with T2DM are associated with increased risks of late tumor stage like TNBC, larger tumor size, and invasive lymph nodes at the time of breast cancer diagnosis [1]. A large, US cohort, prospective sister study of 44,541 female participants diagnosed with T2DM and breast cancer reported the impact of metformin use on the reduced risk of ER^+^ breast cancer but an increased risk of developing TNBC [19]. Due to the complications in detecting TNBC, recommendations addressed to the researchers to further explore the breast screening in women with T2DM through biomarker discovery and physicians are urged to be aware of enhanced risk of late-stage cancers, larger tumor sizes, and more lymph node invasion in women with T2DM [1].

## 6. Biomarkers and Therapies

### 6.1. Circulating Biomarkers

#### 6.1.1. Glycosylated Hemoglobin (HbA1c)

Glycemic control based on glycosylated hemoglobin (Hgb) A1c measurements indicate the three categories of glycemic control: well controlled (HgbA1c < 7.0), moderately controlled (HgbA1c 7.0–9.4), or poorly controlled (HgbA1c ≥ 9.5). TNBC incidence increases with worsening glycemic control in T2DM patients [14].

There is also an urgent need to identify potential novel circulating plasma biomarkers, including micro-RNA (miRNA) and long non-coding LncRNA, circulating tumor DNA (ctDNA), metabolites, and circulating tumor cells and exosomes as non-invasive screening for TNBC diagnosis in T2DM patients.

#### 6.1.2. Cell-Free miRNAs and LncRNAs

The cell-free miRNAs, a large family of small 20–22 nucleotides, and LncRNAs, longer than 200 nucleotides, are both considered non-coding RNAs, which play a pivotal regulatory role at the post-transcriptional level leading to either mRNA degradation or translational suppression of more than 30% of genes in the human during development [136,137]. Recently, circulating plasma miRNAs and LncRNAs, considered promising biomarkers in breast cancer, are highly stable in biological fluids and could potentially serve as a liquid biopsy for the real-time evaluation of tumor status. Numerous studies have recently reported miRNA and LncRNA expression profiles strongly associated with TNBC molecular pathogenesis and cancer progression [138,139,140,141,142,143]. Another study showed that expression levels of miR-124a and miR-30d were the highest in the blood of breast cancer patients with T2DM compared to the T2DM patients and breast cancer patients and the lowest in the healthy group, indicating that miR-124a and miR-30d could contribute to the development of breast cancer in patients with T2DM [144]. In addition, found to be upregulated in the plasma of T2DM patients, high serum LncRNAE330013P06 expression levels were also associated with breast cancer progression, family history, and lymph node metastasis compared with non-T2DM patients, T2DM patients without breast cancer, and healthy donors [145]. However, so far, no circulating miRNA and LncRNA expression profiling has been correlated to TNBC in T2DM patients, in comparison to TNBC patients and T2DM patients. However, a recent study revealed that diabetes-regulated, anti-inflammatory LncRNA (DRAIR), characterized as a critical player in T2DM patients, was overexpressed in patients’ TNBC tissues and was associated with chemoresistance and tumor recurrence of TNBC cell lines under doxorubicin treatment [146].

#### 6.1.3. Circulating Tumor DNA and Cell-Free Nucleic Acids

Blood-based circulating tumor DNA (ctDNA) and cell-free nucleic acids have been known as “liquid biopsies”, referring to alternative biomarkers in the biofluids, which avoid the invasive procedure of tissue biopsies [147,148,149]. ctDNA represents the fraction of circulating free DNA (cfDNA) deriving from cancerous cells and tumors. Several studies have suggested that ctDNA comes from the fragmentation of apoptotic processes that release DNA fragments of between 180–200 bp and necrotic, which give longer DNA fragments due to random digestion of the genomic DNA [149]. ctDNA in the bloodstream of cancer patients contains important information on genetic alterations, including mutations, copy number changes, and epigenetic profiles such as methylation patterns related to cancer development, and progression and drug response which could help detect earlier stages of malignancies and drug response prediction [150,151]. The amount of ctDNA varies among individuals and depends on the type and site of tumor [152]. The analysis of ctDNA during patient follow-up is more practical and less invasive than tissue biopsy, which detects both tumor remission and metastasis that allows for rapid intervention through identification of tumor specific (somatic) variations in the ctDNA [153]. One of the major reasons for the failure of cancer systemic therapies is our inability to accurately capture the heterogeneity of TNBC. There is a longstanding hope of precision medicine to find out genetic markers that can tackle challenges due to inter-individual variability in drug response and early prediction of tumor metastasis using advances in whole-genome and targeted NGS techniques. There is a compelling analysis of circulating cell-free tumor nucleic acids widening TNBC heterogeneity molecular characteristics [154]. In regard to T2DM, a panel of circulating nucleic acid-based biomarkers of T2DM has been reported, including mainly inflammatory-related genes [155]. Liquid biopsy is a tool of precision medicine, which could provide a quick assessment of T2DM patients with BC at early-stage diagnosis, tumor progression monitoring after therapies through the analysis of gene mutations on ctDNA [156]. Recent studies have reported the beneficial interest of ctDNA detection in order to predict recurrence of TNBC patients after neoadjuvant chemotherapy and for prognosis [157,158]. However, until now, no studies have been conducted for the detection of ctDNA in TNBC patients with T2DM.

#### 6.1.4. Exosomes

The exosomes are nanosized lipid bilayer extracellular vesicles, which serve as a cargo containing a complex of mixtures including various types of nuclei acids (miRNA, LnRNA, and DNA) and signaling proteins that are released after binding to recipient cells. An increasing body of evidence revealed the multiple exosome biological functions in TNBC tumorigenesis processes, including tumor initiation, metastasis, drug resistance, and immune escape [159]. In addition, exosomes have been reported as valuable biomarkers for TNBC diagnosis, prognosis, monitoring, and as an engineered delivery vehicle for TNBC treatment [160,161]. In the context of T2DM increasing the risk of lymph node metastasis, an in vitro study reported that T2DM promoted breast cancer metastasis through the resulted functional alterations of the human mesenchymal stem cells (MSCs)-derived exosomes associated with excess secretion of IL-6 from MSCs and the JAK/STAT3 pathway in breast cancer cells [162]. Exosomes-derived primary breast adipocytes have been recently reported to induce immune exhaustion characterized by the reduced effector functions and elevated expression of immune checkpoints (i.e., PD-L1) on multiple T cell subsets in the periphery and tumor infiltrates, which would help evaluate patient responses to checkpoint therapies such as atezolizumab [163].

#### 6.1.5. Metabolomics

The metabolomics can provide clinically valuable information about metabolic alterations, including metabolic reprogramming, in a cancer state requiring large supplies of macro-nutrients and can lead to new insights into the pathology of TNBC and the discovery of potential therapeutic targets aiding in the development of effective therapies [164]. Recently, the use of the NMR spectroscopy has established metabolomic analysis and the construction of a prediction model based on metabolic phenotypes of the response of TNBC patients to neoadjuvant chemotherapy [165]. Three main metabolic pathways of glycine, serine, and threonine metabolism; valine, leucine, and isoleucine biosynthesis; and alanine, aspartate, and glutamate metabolism were revealed to distinguish groups of TNBC patients with no, partial, or complete response [165]. Studies to identify plasma metabolites significantly altered in TNBC patients are scarce and controversial. For example, Wojtowicz et al. [166] observed lower levels of glutamate and higher glutamine levels in the sera of TNBC patients compared with the levels in healthy controls, while Cao et al. [167] observed higher levels of glutamic acid and lower glutamine levels in TNBC tissues. The concept of TNBC glutamine addiction promoting glucose oxidation was also reported [168]. In addition, two studies showed lower levels of valine and tyrosine in the blood of breast cancer patients than in the blood of healthy controls, while the opposite findings were reported in another study [169,170]. So far, there is no metabolomics study reporting the identification of plasma metabolites that significantly change in T2DM patients diagnosed with breast cancer compared to those without diabetes or to healthy controls. Metabolic alterations in TNBC caused by the lack of hormonal receptor expression in breast cancer progression can provide a better understanding of the biochemical changes underlying the different TNBC subtypes.

#### 6.1.6. Circulating Tumor Cells

The circulating tumor cells (CTCs) represent another feature of biomarkers as these tumor cells have been detached from the primary tumor or even from metastatic tumors, shed into the vasculature or lymphatic system and found disseminated in the patient’s blood circulation. Many studies have evidenced CTC presence in TNBC patients at different stages (i.e., early and metastatic settings) with the detection of enhanced nuclear PARP-1 activity and observed an increased enumeration of CTCs in a metastatic advanced stage of TNBC and a drop of CTC percentage after neoadjuvant chemotherapy [171,172]. At an early stage of breast cancer, CTCs can generate micrometastases and serve as surrogates for minimal residual disease (MRD). The detection, characterization, and profiling of these CTCs provide a non-invasive and valuable screening “liquid biopsy” tool for early-stage breast cancer and TNBC [173,174]. From 32 TNBC patients, CTC population was isolated and characterized based on epithelial–mesenchymal transition (EMT) protein expression, a phenotype reflecting the spatiotemporal heterogeneity of the tumor and relevant phenotype for efficient tumor dissemination, and the defined CTC signature demonstrated a prognostic value for the prediction of the patient’s outcome or pathological response [173]. A recent phase 3 randomized STIC CTC trial (NCT01710605) clinical study revealed CTC count as a reliable biomarker after observing an improved overall survival (OS) based on CTC count as a guide first-line cyclin-dependent kinase (CDK)4/6 inhibitors treatment, with chemotherapy or endocrine therapy over the physician’s choice of CTC count for patients with HR^+^/HER2^−^ breast cancer [175]. A retrospective study recruiting 264 Chinese patients with postoperative stage I-III breast cancer has reported the increased CTC count in non-metastatic patients, suggesting the potential association between higher prevalence of CTCs and metabolic dysfunction such as hyperglycemia and a high level of high-density lipoprotein (HDL) in breast cancer patients [176]. Of note, HDL isolated from fasting plasma of T2DM breast cancer patients was demonstrated to promote tumor cell adhesion to vasculature endothelium by inducing overexpression of cell adhesion molecules ICAM-1 and VCAM-1 through protein kinase C signaling pathway activation [177].

## 7. Tissue Biomarkers

In this review, current and potential tissue biomarkers of TNBC with metabolic or pathological functions related to T2DM, including their impact in T2DM/TNBC patients, are mentioned in Table 2.

### 7.1. BRCA1/2

While BRCA1 has been reported for its metabolic actions, *BRCA1* and *BRCA2* germline mutations lead to impaired cell cycle and DNA repair, which represent 70–80% and 16–23% of all TNBC cases [70,178]. A recent case–control study reported that white blood cell *BRCA1* promotes methylation, causing downregulation of the gene expression profile due to homologous recombination deficiency (HRD) and was associated with elevated risk of developing TNBC [179]. Clinicopathological studies with genetic data analysis displayed a higher risk for *BRCA1* mutation carriers of developing TNBC than *BRCA2* mutation carriers do [180,181]. These *BRCA1/2* germline mutations enhance TNBC sensitivity to DNA-damaging agents, resulting in a prolonged survival of these carriers diagnosed with TNBC [181]. From 57 participating centers in seven countries examining 6052 women, it was reported that in the 15-year period after breast cancer diagnosis, the risk of diabetes doubled among *BRCA1/2* carriers compared with carriers without cancer, especially for women with high BMI [69].

### 7.2. Epidermal Growth Factor Receptor

The epidermal growth factor receptor (EGFR) family (also known as HER or erythroblastic leukemia viral oncogene homolog ERBB) constitutes a group of transmembrane protein receptors classified into a subfamily of four closely related ErbB receptor tyrosine kinases (EGFR1/ErbB1, HER2/ErbB2, Her3/ErbB3, and Her4/ErbB4) for specific extracellular protein ligands such as EGF or even transforming growth factor α (TGFα) [182]. Upon activation by its growth factor ligand, EGFR undergoes a transition from an inactive monomeric form to an active heterodimer, resulting in autophosphorylation of several tyrosine residues in the cytoplasmic domain and leading to the activation of several signal transduction pathways, including the phosphatidylinositol-3 kinase (PI3K)/protein kinase B (Akt)/mammalian target of rapamycin (mTOR) pathway, the RAS/RAF/mitogen-activated protein kinase (MAPK) pathway, and the JAK/STAT pathway [183]. These activated signaling pathways stimulate tumor onset, tumor development through increased cell proliferation and inhibition of apoptosis, and tumor progression through increased cell migration, adhesion, invasion and angiogenesis [183]. In physiological situations, EGFR is crucial for ductal development in mammary glands and its ligands amphiregulin, TGFα and heregulin induce both ductal and lobuloalveolar development even in the absence of estrogen or progesterone [184]. In up to 70% of TNBC cases, EGFR1 is commonly overexpressed, revealing EGFR1 as an important diagnostic marker associated with a poor prognosis and in 25% of the TNBC cases, EGFR1 overexpression is caused by its gene amplification and not due to mutations [185]. In addition, the capacity of G protein-coupled receptor agonists promoting EGFR transactivation and accumulation of nuclear EGFR causes drug resistance in TNBC. Activated EGFR signaling is mainly observed in the BL2 and MSL subtypes of TNBC; however, TNBC remains resistant to anti-EGFR therapies [186]. EGFR is also frequently overexpressed in metastatic TNBC (mTNBC). Related to T2DM development, EGFR is described as regulating pancreatic fibrosis [187]. To overcome resistance to EGFR inhibition, a phase 1 study evaluated the combination treatment of mTNBC patients with EGFR inhibitor erlotinib and anti-diabetic drug metformin, resulting in the inhibition of common signaling pathways MAPK and PI3K/mTOR [188]. This combined treatment was well tolerated in the cohort of mTNBC patients with no dose-limiting toxicities. However, no objective responses including response rate, stable disease rate, and progression free survival was observed [188].

### 7.3. Vascular Endothelial Growth Factor

The vascular endothelial growth factor (VEGF) is the most potent pro-angiogenic growth factor mainly expressed in hypoxic conditions by the hypoxic stress transcription factor hypoxia-inducible factor alpha (HIF-α). VEGF is a family of ligands (VEGF-A, VEGF-B, VEGF-C, and VEGF-D) which are specific to the VEGFR family, and results in the stimulation of endothelial cell proliferation and migration, increase of vascular permeability, and enhances tumor cell extravasation. Predominantly expressed in vascular cells including endothelial and smooth muscle cells, the main actors of the formation of new blood vessels from pre-existent vasculature, VEGF is the main factor regulating metastasis formation. In diabetic conditions, VEGF mainly mediates endothelial dysfunction, including diabetic vasculopathy [189]. VEGF is overexpressed in metastatic TNBC cell variants contributing to the formation of metastases by facilitating TNBC cell intravasation to the bone and to the central nervous system (CNS), including the brain [190,191]. High tumor tissue levels of VEGF in patients with primary operable TNBC were associated with shorter survival times compared to non-TNBC patients [192]. Hence, VEGF is considered as a poor prognostic marker in TNBC patients. VEGF is also considered as an early predictive marker, revealed by the reported monitoring VEGF serum levels in TNBC patients, which were correlated with the patient’s response and pathological complete response (pCR) to neoadjuvant chemotherapy (NAC) and with the patient’s survival [193]. An in vitro study monitoring the protein expression levels of VEGF and its receptor VEGFR2, co-receptor Neuropilin-1, adhesion molecules in TNBC cell lines MDA-MB-231 parental, and metastatic variants to bone and brain MDA-MB-231BO and MDA-MB-231BR in hypoglycemia and hyperglycemia, demonstrated that TNBC progression-related adhesion molecules was linked with overexpression of VEGFR2 in hyperglycemia, while VEGFR2 was sustained and not degraded in hypoglycemia for a rapid switch to its mature functional form upon glucose availability [194]. This study suggested a VEGF-dependent link between TNBC progression and hyperglycemia [194]. In T2DM patients, especially in hyperglycemia conditions, the VEGF-A serum levels were reported to increase while high VEGF-B and VEGF-C levels were strongly correlated with obesity, thus revealing the metabolic roles of VEGFs [195]. At the breast tissue level, the disturbances between adipose tissue and endothelial cells are mainly caused by VEGF, which has the ability to enhance perfusion and insulin delivery in adipose tissues [195].

### 7.4. Nuclear Ki-67 Antigen

Ki-67, also known as MKI67 (Marker of Proliferation Ki-67), is a nuclear antigen detected during cell cycle interphase (G1, S, G2) and mitosis while Ki-67 is absent from the cells in a quiescent phase, making Ki-67 the main cell proliferation biomarker routinely used in clinical practice [196]. The growth rate of any tumor is assessed by IHC staining detecting Ki-67 expression and Ki-67 is a useful parameter to distinguish luminal A from luminal B breast cancer subtypes [197]. Mainly expressed in ER^−^ breast cancer, including TNBC and especially in ductal TNBC, a high Ki-67 expression level is considered as a poor prognostic with predictive potential as it is associated with poor outcomes while high mitotic activity in the tumor shows a very good clinical response to combination chemotherapy [198]. A recent study of 170 patients diagnosed with invasive breast cancer (TNBC and HER2^+^/HR^−^ subtype) were subjected to lipid profiling and IHC staining measuring Ki-67 score [199]. Serum dyslipidemia, characterized by elevated total cholesterol and LDL-C levels and reduced HDL-C and apolipoprotein A1, was strongly correlated with Ki-67 expression levels and indicated a poor prognosis in breast cancer (including TNBC) patients [199]. Another study of 102 cases of T2DM patients diagnosed with breast cancer and 106 non-diabetic counterparts reported higher proportions of local recurrence, lymph node metastasis, and distant metastasis and a significant increase in IGF-1R and Ki-67 in the T2DM breast cancer group than in the non-diabetic breast cancer group [200].

### 7.5. Tumor Suppressor Protein p53

The p53 (also known as tumor protein P53) is a stress-sensitive nuclear tumor suppressor transcription factor protein that plays a vital role in controlling cell cycle, apoptosis (i.e., programmed cell death), and in preserving DNA repair and genomic integrity, which gives it its nickname of “guardian of the genome” [201]. Non-canonical functions of p53 revealed its capability to regulate energy metabolism, lipid, and glucose metabolism, including glucose transporter [202]. Mutations in the *TP53* gene cause loss of functions of cell cycle checkpoint controls and impairment of the DNA damage repair system, promoting genomic instability which lead to uncontrolled cell proliferation with replication of damaged DNA replication, resulting in cancer onset, development, and in cancer progression by stimulating invasion and metastasis [203,204]. *TP53* mutations are frequently detected in 65 to 80% of the cases of TNBC patients compared to in luminal breast cancer cases (12%) and were associated with poor prognosis, suggesting p53 as a biomarker [205,206]. Although most of the clinical studies have not found any correlations between *TP53* mutations scoring in patients’ TNBC tissues and patients’ response to conventional neoadjuvant chemotherapy, a recent precision oncology-related study using the Evolutionary Action Scoring system reported the strong association between *TP53* mutations and worst outcomes among 96 TNBC patients with residual disease following chemotherapy [207]. Experimental data provided the evidence of the increase of *TP53* mutations in hyperglycemic conditions and a potentiation of p53 mutants of insulin-induced AKT1 activation by binding and inhibiting the tumor suppressor disabled homolog 2-interacting protein (DAB2IP) in the cytoplasm [208]. Recently discovered to be a genetic risk factor for T2DM (i.e., *TP53* Pro72Arg polymorphism) from a case–control study performed on a Chinese population, the p53 serum level was determined to be strongly correlated with clinical and biochemical parameters in 225 T2DM in comparison to 255 non-diabetic control individuals [209,210].

### 7.6. Nuclear Enzyme Poly(ADP-ribose)polymerase

Poly(ADP-ribose)polymerase (PARP) is a nuclear enzyme that plays a prominent role in the catalysis of the ADP-ribose units polymerization, units derived from the ADP donor nicotinamide adenine dinucleotide (NAD)+. PARPs constitute a family of eukaryotic enzymes responsible for the maintenance of cellular homeostasis through their involvement in various biological processes, including DNA damage repair (homologous recombination and non-homologous end-joining-based repair mechanisms), transcription, genomic stability, chromatin structure modulation, recombination, cell replication, cell adhesion and motility, innate immunity, and transformation [211]. At the metabolic level, PARP activation exhibits a disruptive signal to lipid metabolism and contributes to insulin resistance [212]. By its particularity as a DNA damage nick sensor enzyme, PARP1, the most common PARP isoform, repairs DNA single-stranded breaks (SSB) through base excision repair (BER) by bringing crucial enzymes required for DNA repair after its binding to the exposed ends of the damaged DNA strand [211]. Failure of the BER pathway results in the accumulation of DNA SSBs, which compromises the DNA replication and transcription process, leading to genome instability and even cell death through induction of apoptosis [213]. Both BRCA1 and BRCA2 operate DNA repair through homologous recombination (HR) and non-homologous end joining (NHEJ) systems for the repair of DNA double-stranded breaks (DSBs) and through BER pathways like PARP for the repair of DNA SSBs [214,215]. Hence, the loss of functions of both BRCA and PARP results in the cancer cell death whereas their individual loss of functions is not lethal, which describes their genetic interactions as “synthetic lethal” [216]. Thus, PARP inhibition caused by FDA-approved PARP inhibitor (PARPi) drugs (e.g., olaparib, iniparib) potentiate the effects of DNA-damaging agents and ionizing radiations in the therapy of TNBC, especially BRCA-associated breast cancer [217,218,219]. A clinical study reported that (61) T2DM subjects with poor glycemic control presented higher levels of peripheral blood mononuclear cells’ (PBMCs) nuclear extract-derived poly(ADP-ribose), a surrogate of PARP activity, than (48) normoglycemic individuals [220]. This PARP hyperactivation was strongly associated with high HbA1c levels and with the dysregulation of DNA methylation [220]. An experimental study demonstrated the TNBC cells’ resistance to PARPi, characterized by EMT and PD-L1 upregulation, was reversed by the anti-diabetic drug metformin through the blockade of the PI3K/AKT survival pathway [221]. This study suggested a promising approach of a combined treatment metformin and PARPi to enhance PARPi efficacy and tumor sensitivity to immunotherapy [211]. This promising approach could be beneficial for T2DM patients diagnosed with breast cancer, including TNBC.

### 7.7. mTOR/Ribosomal Protein (p70) S6 Kinase 1 Pathway

**Figure 1:**
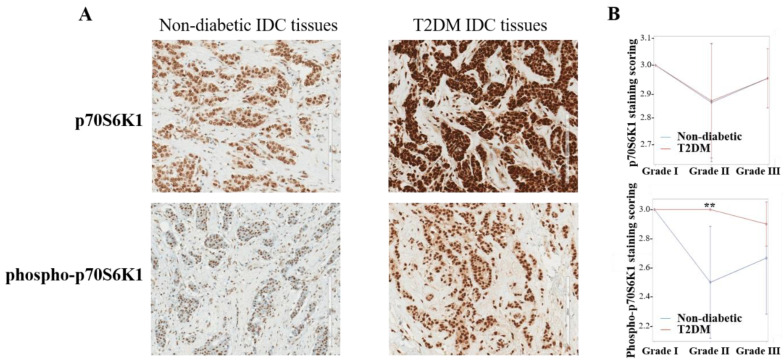
(**A**) Representative IHC staining of total p70S6K1 and phospho-p70S6K1 in grade II invasive ductal carcinoma (IDC) extracted from T2DM and non-diabetic patients showing a weak expression of phospho-p70S6K1 in non-diabetic IDC tissues while phospho-p70S6K1 was overexpressed in T2DM IDC tissues. Scale bar: 200 μm. (**B**) Scoring of phospho-p70S6K1 and p70S6K1 expression in IDC tissues based on the grade of the breast cancer tissues of T2DM and non-diabetic patients. ** *p* < 0.01 was obtained using ORTHOREG procedure from SAS system software (version 9.2, SAS Institute Inc., Cary, NC, USA) with post-hoc Tukey Honestly Significant Difference test.

The ribosomal protein (p70) S6 Kinase 1 (p70S6K1) is the downstream effector of lipid kinase PI3K/Akt/mTOR complex 1 (PAM), one of the most important and active pathways in breast cancer including TNBC and in metabolic alterations such as T2DM [222,223]. The p70S6K1, upon its activated form phospho-p70S6K1, contributes to additional lipid production and protein synthesis, and to cell survival through cell cycle progression, tumorigenesis, tumor angiogenesis, and chemotherapeutic drug resistance at the cellular level [224]. Mainly caused by PTEN loss-of-function, hyperactivation of the PAM pathway is associated with poor prognosis and chemoresistance in TNBC patients [225]. A clinical study reported the influence of pathogenic factors of T2DM on the activation of the PAM pathway, resulting in p70S6K1 phosphorylation detected in peripheral blood mononuclear cells (PBMCs) collected from postmenopausal healthy women, T2DM women, women diagnosed with endometrial and breast cancer, and from T2DM postmenopausal women diagnosed with endometrial and breast cancer [226]. Additionally, we reported that glycated albumin, belonging to the heterogeneous group of advanced glycation end-products (AGEs), increased the phosphorylation of p70S6K1 (phospho-p70S6K1) 25-fold within 10 min in a highly invasive breast cancer MDA-MB-231 cell line (a MSL TNBC model) while most of the signaling protein phosphorylation enhanced by 5–7.5-fold, suggesting phospho-p70S6K1 as a potential biomarker for breast cancer progression in diabetic conditions [227]. To verify this diabetes-induced p70S6K1 over-phosphorylation, a retrospective study consisting in IHC staining of both phospho-p70S6K1 and total p70S6K1 protein expression was performed using invasive ductal carcinoma (IDC) FFPE tissues extracted from 16 postmenopausal T2DM and 16 non-diabetic patients enrolled in 2016–2017 at the Oncology Division, King Abdullah Medical City, Riyadh, Saudi Arabia. Written informed consent was obtained from all patients. Based on the scoring, a parameter assessing the degree of the protein expression, the phospho-p70S6K1 was highly expressed in IDC tissues of T2DM patients as compared with phospho-p70S6K1 expression level weakly detected in IDC tissues of non-diabetic patients (Figure 1A). A high level of phospho-p70S6K1 expression in T2DM conditions compared to non-diabetic counterparts was revealed to be significant (*p* < 0.005) in grade II presenting ER^+^/PR^+^/HER2^−^ and not in grade I or even in advanced grade III (Figure 1B). As for the difference of its phospho-form, the expression level of the total form of p70S6K1 did not change between non-diabetic and T2DM breast cancer tissues (Figure 1). Vatseba and colleagues observed that in collected PBMCs the highest level of p70S6K1 phosphorylation was determined in the T2DM group, while an unexpected result of reduction of p70S6K1 phosphorylation was noticed in postmenopausal T2DM patients diagnosed with cancer [226]. Our current data revealed the over-phosphorylation of the ribosomal p70S6K1 intensely detected in IDC tissues of diabetic women compared to those observed in their non-diabetic counterparts’ IDC tissues, suggesting phospho-p70S6K1 as a promising therapeutic target against invasive breast cancer diagnosed in T2DM patients. Further IHC staining of phospho-p70S6K1 vs. total p70S6K1 is still required to be confirmed in a larger cohort.

**Table 2:** TNBC biomarkers with potential metabolic functions and their impact on T2DM/TNBC patients.

Gene	Main Cellular Functions	Metabolic/Pathological Functions Related to T2DM	Types of Alterations in TNBC	Impact on T2DM/TNBC Patients
**BRCA 1/2**	Essential role in DNA repair mechanisms [178]	BRCA1-induced metabolic reprogramming of breast cancer cells, glycolysis inhibition, complex interactions with insulin/insulin-like growth factor (IGF-1) signaling axis [70]	Loss of function mutation, Epigenetic changes [178]	Increased risk of diabetes in BRCA1 or BRCA2 mutation carriers within 15 years after diagnosis of TNBC [69]
**EGFR**	Stimulation of cell proliferation, migration, apoptosis, adhesion, invasion, and differentiation [183]	Regulating pancreatic fibrosis [187]	Overexpression due to gene amplification [185]	Used as diagnostic marker associated with poor prognosis in 70% of TNBC cases [185]
**VEGF**	Enhancing vascular permeability, tumor cell extravasation, cell proliferation and migration [190]	Mediating diabetic endothelial dysfunction, including diabetic vasculopathy [189]	Mutation, overexpression, and amplification [36]	High tumor levels of VEGF reducing survival times in primary operable TNBC [192]. Early predictor of pathological complete response to neoadjuvant chemotherapy and survival in TNBC patients and increased in T2DM patients [193,195]
**Ki-67**	Crucial for perichromosomal layer formation and preventing mitotic chromosome aggregation [196]	No reported metabolic function	Overexpression [44]	Significantly increased in T2DM breast cancer group [200].
**TP53**	Pivotal role in cell cycle arrest, apoptosis, DNA repair, and genomic integrity [201]	Molecular regulation of several key metabolic regulators involved in lipid and glucose metabolism, glucose transporter [202]	Loss of function mutation [36]	TP53 mutations associated with worse outcomes in TNBC patients with residual disease following chemotherapy [207]. High levels of P53 serum correlated with clinical and biochemical parameters in T2DM patients [209,210]
**PARP**	Responsible for DNA damage repair, regulating innate immunity response, transformation, cell adhesion and motility, damage repair, transcription, genomic stability, chromatin structure modulation, recombination, cell replication [211]	Disruptive signal to lipid metabolism, hyperlipidemia development, insulin resistance [212]	High expression of nuclear PARP-1 [172]	Increasing of peripheral blood mononuclear cells (PBMCs) nuclear ADP-ribose, a surrogate of PARP activity, in uncontrolled glycemic cases [220]
**mTOR/** **p70S6K1 Pathway**	Induction of protein synthesis, cell growth, survival, tumorigenesis, chemotherapeutic drug resistance [224]	Crucial role in lipid production, inhibition of mTOR pathway promoting insulin resistance and metabolic alterations, including T2DM [222]	mTOR pathway hyperactivated involved in TNBC survival and chemoresistance [223]	Higher mTOR pathway activation in TNBC, associated with poor prognosis [225]

## 8. Metformin, Anti-Diabetic with Anticancer Therapy and Ongoing Related Clinical Trials

Metformin, chemically known as 1,1 dimethyl-biguanide hydrochorine, is a member of the class of guanidines derived from the plant Galega officinalis (French lilac) and is frequently used as the first-line antihyperglycemic drug to lower glycemic level and manage T2DM without causing hypoglycemia in T2DM subjects. Indicated as an adjunct to diet and exercise, metfomin exhibits various hypoglycemic properties through reduction of hepatic glucose production, decreasing the intestinal absorption of glucose and through enhancement of insulin sensitivity by increasing both peripheral glucose uptake and utilization [228]. However, the use of metformin by T2DM subjects does not change the secretion of insulin, sustaining hyperinsulinemia [229]. Acting mainly inside the hepatocytes with entry facilitated by organic cation transporter-1 (OCT-1), metformin inhibits the mitochondrial complex I activity and subsequently cellular respiration by preventing mitochondrial adenosine triphosphate (ATP) production and increasing the production of cyclic adenosine monophosphate (cAMP) [230]. These metabolic changes activate AMP-activated protein kinase (AMPK), an enzyme known to be a tumor suppressor by regulating downstream anabolic pathways that are crucial for tumor cell proliferation and for fatty acid synthesis [231]. In addition, metformin inhibits oncogenic activation of PI3K/AKT/mTOR signaling, the pathway primarily activated by the insulin receptor, thereby repurposing metformin as an anticancer drug [232,233]. A randomized clinical trial reported the anticancer effects of metformin administered to non-diabetic women with operable invasive breast cancer, which was associated with phospho-AMPK upregulation, phospho-AKT downregulation, and suppression of insulin responses [234]. Regarding the outcome of the administration of metformin to non-diabetic or T2DM patients diagnosed with breast cancer or TNBC, there are many controversies. Several clinical observational studies and a phase 3 randomized trial (i.e., MA.32) reported that the administration of metformin alone (i.e., 850 mg) or combined with first-line neoadjuvant chemotherapeutic agents (i.e., standard chemotherapy or hormone therapy) failed to improve disease-free survival for TNBC or even metastatic breast cancer patients with or without T2DM [21,235]. However, a meta-analysis of pre-clinical studies reported the beneficial effects of metformin in non-diabetic and T2DM patients diagnosed with breast cancer, including TNBC, leading to the reduction of cancer risk and improvement of DFS [236]. These controversies in observational studies may be due to differing metabolic status (i.e., BMI, glycaemia control, insulin resistance) of non-diabetic and T2DM patients, and due to the biological impact of T2DM on TNBC such as delayed breast cancer diagnosis, poor treatment adherence, or other factors. Recently, an epidemiological study reported that a 15-year long-term use of metformin reduced the risk of ER-positive breast cancer of T2DM patients by 39%, while there was a 74% increase in the risk of developing TNBC among T2DM subjects treated with metformin [19]. However, most of the experimental data compiled evidence of the potential beneficial effects of metformin against TNBC and even TNBC stem cells, the main cause of tumor metastasis and relapse. In vitro studies reported that metformin interferes in TNBC development and progression by targeting key metabolic defects in lipid and carbohydrate metabolism [237]. Metformin was also demonstrated to overcome TNBC cell line (i.e., Hs 578T, MDA-MB-231) resistance to cisplatin by downregulating the DNA repair protein RAD51 [238]. Using MDA-MB-231 cells as a TNBC model, metformin combined with resveratrol could effectively prevent TNBC progression by enhancing the reducibility properties of the natural oxidant resveratrol [239]. In addition, metformin has been reported to suppress TNBC stem cells through the inhibition and degradation of the protein kinase A (PKA)/glycogen synthase kinase (GSK)-3β/Krüppel-like factor 5 (KLF5) signaling pathway [240]. An on-going phase 2 clinical trial (NCT04248998, the BREAKFAST Trial) conducted in Italy has been recruiting TNBC patients for the monitoring of the combination of chemotherapeutic agents and a diet mimicking FASTing plus/minus metformin in the preoperative setting.

## 9. Conclusions and Perspectives

T2DM subjects have an increased risk of developing breast cancer, especially TNBC, a fast growing and aggressive malignant tumor usually presenting poor clinical outcome due to the lack of targeted therapies, which requires to be screened and detected early. This review lists the main clinicopathological features, related risk factors, current and potential biomarkers for clinical screening, diagnosis, prognosis, monitoring, and development of novel therapies including immunotherapies, and ongoing clinical trials for TNBC patients with T2DM. So far, immunotherapies intensively implemented in clinical trials to improved TNBC outcome require challenging molecular and cellular investigations for T2DM patients with exhausted immune systems and dysfunctional tumor microenvironments [241,242]. Risk factors related to the lifestyle of T2DM subjects have had an important impact on TNBC risk, making physical activity and healthy diet important to lower body fat, hormonal levels, and reduce inflammation, which subsequently decrease TNBC risk. Deeper investigation for the identification of polygenic risk factor or genetic polymorphisms predisposed to the TNBC risk in T2DM subjects would facilitate preventive medicine and provide new insight into how T2DM-related genes affect TNBC risk, which may reduce the high burden of TNBC in T2DM patients. There is still a need to identify more circulating plasma biomarkers for non-invasive screening of TNBC in T2DM for a better management and monitoring of TNBC development and progression in response to the anticancer therapy. Multiple metabolic abnormalities and dysregulated signaling pathways contributing to T2DM development and to TNBC development and progression (i.e., oncogenic markers playing a metabolic regulator role such as BRCA, p53, mTOR/p70S6K1) should be investigated intensively in order to design tailored therapy and personalized strategies for an improvement of T2DM patients diagnosed with TNBC.

## Data Availability

The dataset analyzed during the p70S6K1-related study are available from the corresponding author on reasonable request.
